# Characterising generalism in clinical practice: a systematic mixed studies review protocol

**DOI:** 10.3399/BJGPO.2021.0029

**Published:** 2021-06-16

**Authors:** Martina Kelly, Sarah Cheung, Mariam Keshavjee, Anna Stevenson, Josephine Elliott, Surinder Singh, Madeleine Foster, Sophie Park

**Affiliations:** 1 Department of Family Medicine, Cumming School of Medicine, University of Calgary, Calgary, Alberta, Canada; 2 School of Clinical Medicine, University of Cambridge, Cambridge Biomedical Campus, Cambridge, UK; 3 Research Department of Primary Care and Population Health, University College London, Royal Free Campus, London, UK

**Keywords:** general practice, general practitioners, physicians, family, generalism, generalists, primary health care, secondary care, physicians

## Abstract

**Background:**

Generalist physician care is associated with improved patient outcomes. Despite initiatives to promote generalism in educational settings, recruitment to generalist disciplines remains less than required to serve societal needs. Increasingly this impacts not just general practice but also generalist specialties such as internal medicine, surgery, and paediatrics. One potential factor for this deficit is a lack of explicit attention to generalism as a praxis, including clarifying key aspects of generalist expertise.

**Aim:**

To examine empirical clinical literature on generalism, and characterise how generalism is described and delivered by physicians in primary and secondary care.

**Design & setting:**

A systematic mixed studies review (SMSR) including quantitative, qualitative, mixed-methods studies, and systematic reviews of physician generalist practice.

**Method:**

MEDLINE, Psycinfo, SocINDEX, Embase, Ovid HealthSTAR, Scopus, and Web of Science will be searched for English language studies from 1999 to present, using a structured search. Given study heterogeneity, quality appraisal will not be performed. Two reviewers will perform study selection for each study. Data extraction will focus on how generalism is defined and characterised, including the clinical care provided by generalists and patient experiences of generalist care. Quantitative and qualitative data will be summarised in tabular and narrative form. Convergent synthesis design will then be used to synthesise quantitative and qualitative data.

**Conclusion:**

Findings will characterise generalism and generalist practice from a grassroots clinical perspective. By identifying similarities and differences across generalist disciplines, this work will inform more focused educational initiatives on generalism at undergraduate and postgraduate level, including collaborations between generalist disciplines.

## How this fits in

Despite advocacy for generalism by professional bodies, recruitment to generalist specialties is low. One potential reason is a lack of clarity on key features of generalism and how they overlap or potentially differ across disciplines. Understanding how generalism is characterised in the clinical physician literature offers insights into how generalism is practised in different clinical disciplines. This information can be used to tailor educational initiatives to promote generalist skill sets.

## Introduction

### Background and rationale

A robust workforce of GPs is described as ‘the backbone’ or ‘cornerstone’ of health systems, which enables effective delivery of health care when working collaboratively with specialist colleagues.^1,2^ Benefits of having more GPs include improved patient health outcomes and efficient cost-effective healthcare systems.^3,4^ Yet recruitment into generalist specialties in many developed countries, including the US, UK, and Canada is less than anticipated to meet societal healthcare needs.^5–7^

Professional bodies have repeatedly endorsed training in generalism across the specialties^2,8–11^ and equipping graduates with a generalist skill-base is a key target outcome at an undergraduate level.^12,13^ Health systems have also established targets and programmes to attempt to increase the number of generalist-trained doctors, such as the NHS Long Term Plan in the UK,^14^ which emphasises both the need to develop generalist skills as well as specifically aiming for more GPs. In Canada there is an expectation that at least 40% of graduates will enter family medicine,^6^ while in the UK, Health Education England (HEE) has mandated that 50% of graduates should enter general practice training.^15^ Yet despite multiple efforts in both countries at undergraduate and postgraduate level to support these targets, recruitment is still less than required to meet system needs. In the US, which has traditionally relied more heavily on a specialist workforce, recruitment into generalist specialties, such as general internal medicine, is also less than anticipated to meet population needs.^16^

A key difficulty educating about generalism is the concept itself. Different disciplines offer varying definitions for generalism and emphasise different characteristics of generalist practice.^2,6^ Learners may experience generalist practice in a range of clinical disciplines, but there is rarely explicit attention to the underpinning constructs or skills that constitute generalism. Traditional portrayals of generalist practice frequently focus on general practice (or family medicine), yet there is also a growing emphasis on generalism in specialty disciplines, which increasingly differentiate between ‘generalist’ specialties (for example, internal medicine, surgery, and paediatrics) and subspecialty practices. Thus, there is a growing awareness that generalist practice is not limited to general practice, but instead can — and needs to — be practised across medical disciplines.^1,14^

### Aim

To develop curricula that concretely promote generalism and inform patient care, the authors propose a need to more clearly articulate and characterise generalism and generalist practice. The aim of this review is to identify, describe, and synthesise characteristics of generalism as practised by physicians in the clinical literature. The hypothesis is that by starting at a ‘grassroots’ level, areas of overlap and difference in how different disciplines conceptualise generalism will be able to be ascertained and ‘doing’ generalism will be described.

## Method

### Search strategy

Systematic searches will be conducted in MEDLINE, Psycinfo, SocINDEX, Embase, Ovid HealthSTAR, Scopus, and Web of Science. Search terms will include ‘generalism’, generalist’, and terms representing general practice. To ensure representation from generalist specialty disciplines, generalism search terms will also be combined with ‘internal medicine’, ‘surgeon’, ‘paediatrics’, and ‘psychiatry'. The detailed search strategy is provided in Table 1. The final search will be reported using the PRISMA flowchart.^17^

**Table 1.** Sample search strategy for Ovid MEDLINE

1. Generalism*.tw,kf. (292)
2. generalist*.tw,kf. (8524)
3. 1 or 2 (8686)
4. general practice/ or family practice/ (73 124)
5. Internal Medicine/ (17 299)
6. General Surgery/ (37 803)
7. Psychiatry/ (38 399)
8. Pediatrics/ (51 048)
9. ("general practice*" or "family practice*" or "family medicine").tw,kf. (56 709)
10. "internal medicine*".tw,kf. (23 076)
11. "general surgery".tw,kf. (10 266)
12. psychiatry.tw,kf. (52 000)
13. (pediatrics or paediatrics).tw,kf. (40 002)
14. Physicians, Family/ (16 003)
15. physician*.tw,kf. (367 836)
16. (doctor or doctors).tw,kf. (114 122)
17. surgeon*.tw,kf. (183 634)
18. "general practitioner*".tw,kf. (47 513)
19. psychiatrist*.tw,kf. (23 800)
20. (pediatrician or paediatrians).tw,kf. (5610)
21. 4 or 5 or 6 or 7 or 8 or 9 or 10 or 11 or 12 or 13 or 14 or 15 or 16 or 17 or 18 or 19 or 20 (927725)
22. 3 and 21 (2348)
23. limit 22 to english language (2115)
24. limit 23 to yr="1999–2019" (1431)

### Study selection criteria

Studies from all countries will be eligible; however, only studies in the English language published between 1999 and the present will be included. Any empirical study design that uses primary and secondary evidence (quantitative, qualitative, mixed-methods studies, and systematic reviews) will be included. Conceptual articles, policy articles, conference abstracts, theses, books, general discussions, or letters will be excluded; however, they may be used to identify related studies. Forward-citation searches and hand-searching the references of relevant documents will also be undertaken.

### Screening and data extraction

Study titles and abstracts will be reviewed for eligibility independently and in duplicate by two reviewers. If insufficient information is available to determine suitability, the full text will be retrieved. Disagreements regarding inclusion will be resolved by discussion with the research team. A data extraction form will be piloted and adapted as required. Data extraction processes will be standardised between members by comparison and discussion. Key study information will include: study title; name of first author; year of publication; country of study; study type (qualitative, quantitative, mixed-methods studies, or systematic review); study context (primary, secondary care, or a mixture of both); study population; study aim; and key findings (outcomes), including patient experiences. Introduction and discussion sections of included articles will be examined for definitions of generalism and text describing generalism.

### Quality appraisal and data synthesis

**Figure 1.**
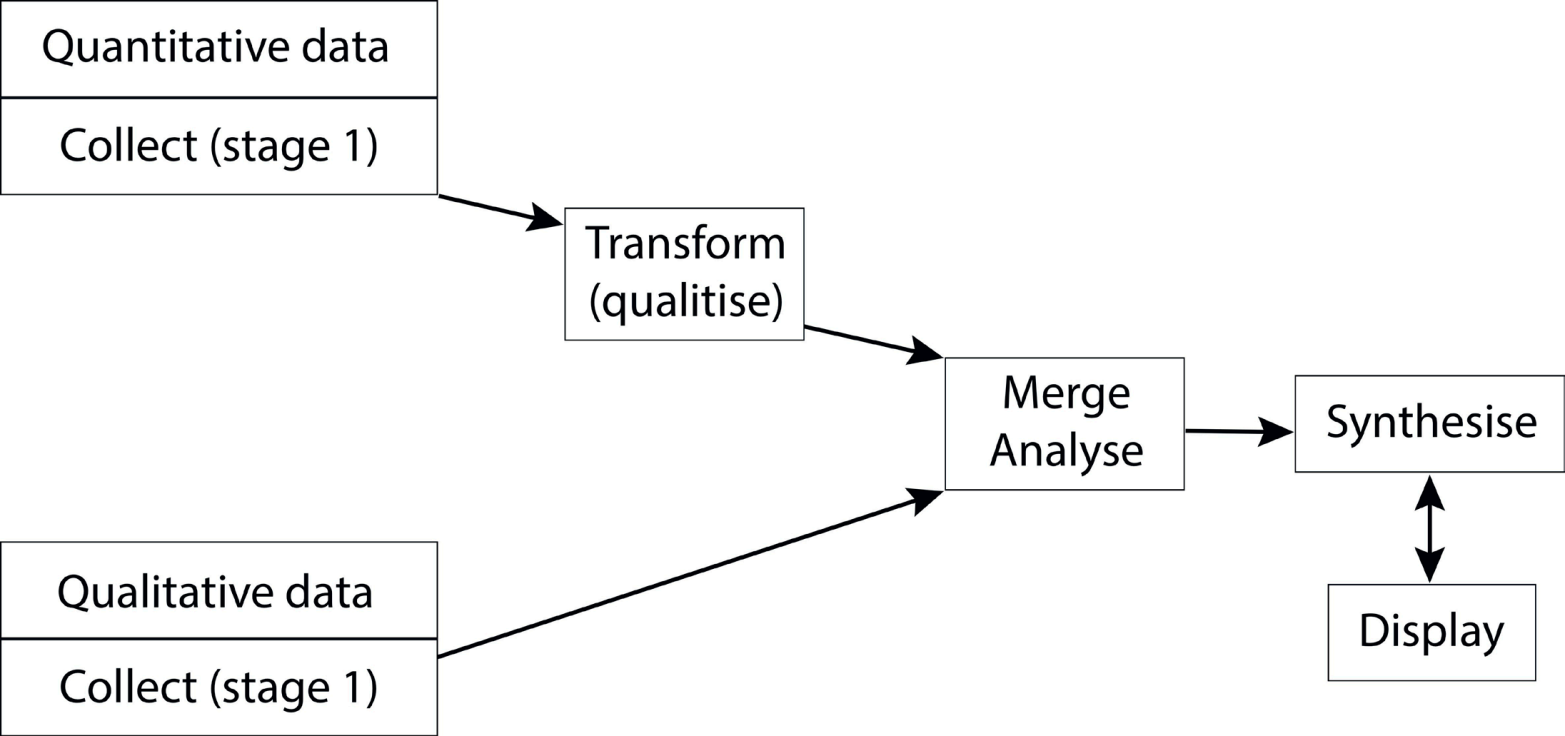
Overview of data extraction and synthesis

A wide range of critical appraisal tools have been developed in recent years to assess mixed-methods research, including systematic mixed-methods reviews.^18^ Despite this, consensus on how best to evaluate study quality for mixed-methods studies is lacking, particularly in relation to qualitative studies included in mixed-methods reviews.^19–21^ Quality appraisal is usually conducted to check the trustworthiness of individual studies in a review and whether the quality may impact review findings.^20^ In this SMSR the focus will not be on the findings of each study per se, but rather on generating a better understanding of how generalism is articulated in clinical practice. To maximise the range and breadth of included articles, methodological quality of included studies will not be assessed.^22^ Study characteristics will be tabulated and described using descriptive statistics, then transformed into textual categories. Template analysis will be used to integrate and explore relationships in the data.^23,24^ Central to the technique is the development of a coding template, which is then applied to data and revised as necessary. The initial template will be devised based on *a priori* concepts of generalism commonly found in the literature, such as patient-centred care, broad knowledge base, collaboration, continuity, and complexity.^1,10,25–27^ This template will be applied tentatively to a subset of data, then refined, including adding new themes as identified. Findings will be aggregated using a convergent design approach, whereby all data are analysed using the same synthesis method and presented together in a joint display (Figure 1). Joint displays of data in health sciences mixed-methods research enable a visual means by which new insights and inferences from integration of the data are possible.^28^

### Patient and public involvement

To ensure attention to patient perspectives, a subset of studies that include patient participants will be identified. Patient characteristics, the nature of patient experiences reported, and engagement with generalist health services will be synthesised. Patients will be invited to comment on review findings.

### Amendments

Any amendments to this protocol will be documented and reported in the final manuscript.

### Dissemination

The results of this review will be published in a peer-review journal and disseminated in various media, including presentations at generalist clinical and medical education conferences, and through research group websites. Input will also be invited from a patient experience group to inform dissemination to public audiences.

## Discussion

### Summary

While generalist practice is endorsed by professional bodies and healthcare policy, recruitment into generalist disciplines is less than required to deliver high-quality patient care. This is no longer a challenge for general practice alone, but increasingly one encountered in generalist specialist disciplines. Generalism and general practice are not synonymous^7^ but likely represent a continuum where different disciplines emphasise different aspects of generalism. This study aims to review and synthesise key features of generalism, as characterised in the clinical literature, with a view to mapping areas of overlap and variance in the practice of generalism.

### Strengths and limitations

Strengths of this review include its broad search and use of a mixed-methods approach to include all relevant empirical study designs to produce insights greater than reported with quantitative and qualitative findings that are separated.^11,22,29–31^ Studies from all countries are eligible for inclusion and the international team includes learners, researchers, and clinicians, which will promote critical discussions on the interpretations. A limitation of this protocol is that the team is composed of physicians and researchers based in general practice. Therefore, the focus is on physician practice only. Generalists also include other healthcare professionals, such as nurse practitioners, and so future work that investigates the articulation of generalism in other healthcare professionals may provide complementary insights.

### Comparison with existing literature

A number of reviews of generalism are reported in professional statements on generalism,^1,2,10,23,32,33^ but these are not reported as systematic reviews, available in the public literature. While one scoping review has been identifed on generalism in family medicine,^34^ this is confined to rural medicine. As far as the authors are aware, no cross-disciplinary review on generalism is available.

### Implications for research and practice

The findings of this review will allow for more elaborated articulation of generalism, resulting in increased clarity on key features of generalism in practice across disciplines, and identify areas of similarity and difference across different disciplines. This data could be used to develop more refined and targeted curricula for learners at undergraduate and postgraduate level. In addition, focusing on patient characteristics and experiences may offer new insights into how generalism should evolve to maximise patient benefit and embed user perspectives in health care.

## References

[1] PGME Collaborative Governance Council (2018). Report on generalism in postgraduate medical education. https://web.archive.org/web/20200804205437/https://pgme-cgc.ca/sites/default/files/news/Generalism%20Working%20Group%20Position%20Paper%20FINAL.pdf.

[2] Commission on Generalism (2011). Guiding patients through complexity: modern medical generalism. Report of an independent commission for the Royal College of General Practitioners and The Health Foundation. https://www.health.org.uk/publications/guiding-patients-through-complexity-modern-medical-generalism.

[3] Starfield B, Shi L, Macinko J (2005). Contribution of primary care to health systems and health. Milbank Q.

[4] Larson EB, Grumbach K, Roberts KB (2005). The future of generalism in medicine. Ann Intern Med.

[5] Dalen JE, Ryan KJ, Alpert JS (2017). Where have the generalists gone? They became specialists, then subspecialists. Am J Med.

[6] Bosco C, Oandasan I (2016). Review of family medicine within rural and remote Canada: education, practice, and policy. https://www.cfpc.ca/uploadedFiles/Publications/News_Releases/News_Items/ARFM_BackgroundPaper_Eng_WEB_FINAL.pdf.

[7] Wass V, Gregory S, Petty-Saphon K (2016). By choice — not by chance: supporting medical students towards future careers in general practice.

[8] Oliver D (2016). David Oliver: celebrating the expert generalist. BMJ.

[9] Reeve J (2010;). Interpretive medicine: supporting generalism in a changing primary care world. Occas Pap R Coll Gen Pract.

[10] Royal College of General Practitioners (2013). Medical generalism: impact report, May 2013. http://www.rcgp.org.uk/policy/rcgp-policy-areas/medical-generalism.aspx.

[11] Reeve J, Beaulieu M-D, Freeman T (2018). Revitalizing generalist practice: the Montreal statement. Ann Fam Med.

[12] The Association of Faculties of Medicine of Canada (2015). The Future of Medical Education in Canada (FMEC): a collective vision for MD education 2010–2015. https://www.afmc.ca/web/en/projects-resources/future-of-medical-education-of-canada-fmec.

[13] General Medical Council (2018). Outcomes for graduates 2018. https://www.gmc-uk.org/-/media/documents/dc11326-outcomes-for-graduates-2018_pdf-75040796.pdf.

[14] NHS England (2019). The NHS Long Term Plan: easy read. https://www.longtermplan.nhs.uk/wp-content/uploads/2019/01/easy-read-long-term-plan-v2.pdf.

[15] Department of Health (2013). Delivering high quality, effective, compassionate care: developing the right people with the right skills and the right values. A mandate from the government to Health Education England: April 2013 to March 2015. https://assets.publishing.service.gov.uk/government/uploads/system/uploads/attachment_data/file/203332/29257_2900971_Delivering_Accessible.pdf.

[16] Merritt Hawkins (2018). White paper series. Internal medicine recruiting trends and recommendations. https://www.merritthawkins.com/uploadedFiles/MerrittHawkins_InternalMedicine_Whitepaper_2018.pdf.

[17] Moher D, Shamseer L, Clarke M (2015). Preferred reporting items for systematic review and meta-analysis protocols (PRISMA-P) 2015 statement. Syst Rev.

[18] Hong QN, Fàbregues S, Bartlett G (2018). The mixed methods appraisal tool (MMAT) version 2018 for information professionals and researchers. Education for Information.

[19] Hong QN, Pluye P (2019). A conceptual framework for critical appraisal in systematic mixed studies reviews. J Mix Methods Res.

[20] Carroll C, Booth A (2015). Quality assessment of qualitative evidence for systematic review and synthesis: is it meaningful, and if so, how should it be performed?. Res Synth Methods.

[21] Brooks H, Llewellyn CD, Nadarzynski T (2018). Sexual orientation disclosure in health care: a systematic review. Br J Gen Pract.

[22] Hong QN, Pluye P, Bujold M, Wassef M (2017). Convergent and sequential synthesis designs: implications for conducting and reporting systematic reviews of qualitative and quantitative evidence. Syst Rev.

[23] Royal College of Physicians and Surgeons of Canada (2013). Report of the generalism and generalist task force.

[24] Freeman T (2006). Celebrating our role as generalists. Can Fam Physician.

[25] Gofton W, Regehr G (2006). What we don't know we are teaching: unveiling the hidden curriculum. Clin Orthop Relat Res.

[26] Mulder H, Ter Braak E, Chen HC, Ten Cate O (2019). Addressing the hidden curriculum in the clinical workplace: a practical tool for trainees and faculty. Med Teach.

[27] Hafferty FW (1998). Beyond curriculum reform: confronting medicine's hidden curriculum. Acad Med.

[28] Hafferty FW, Franks R (1994). The hidden curriculum, ethics teaching, and the structure of medical education. Acad Med.

[29] Fleming J, Patel P, Tristram S, Reeve J (2017). The fall and rise of generalism: perceptions of generalist practice amongst medical students. Educ Prim Care.

[30] Strasser R, Cheu H (2018). Needs of the many: Northern Ontario school of medicine students' experience of generalism and rural practice. Can Fam Physician.

[31] Draper P, Smits HL (1975). The primary-care practitioner — specialist or jack-of-all trades. N Engl J Med.

[32] Gutkin C (2012). Focusing on generalism. Can Fam Physician.

[33] College of Family Physicians of Canada (2020). Proceedings from the College of Family Physicians of Canada undergraduate education retreat on advancing generalism. https://www.cfpc.ca/cmsadmin/CFPC/media/PDF/2020-Undergraduate-Retreat-Proceedings-EN.pdf.

[34] Schubert N, Evans R, Battye K (2018). International approaches to rural generalist medicine: a scoping review. Hum Resour Health.

